# A Novel Mutation in *Leptin* Gene Is Associated with Severe Obesity in Chinese Individuals

**DOI:** 10.1155/2014/912052

**Published:** 2014-02-23

**Authors:** Yue Zhao, Nanchao Hong, Xiao Liu, Beibei Wu, Shanshan Tang, Jianjun Yang, Cheng Hu, Weiping Jia

**Affiliations:** ^1^Translational Medical Center, Shanghai Diabetes Institute, Department of Endocrinology and Metabolism, Shanghai Key Laboratory of Diabetes Mellitus, Shanghai Jiao Tong University Affiliated Sixth People's Hospital, 600 Yishan Road, Shanghai 200233, China; ^2^Department of General Surgery, Shanghai Ninth People's Hospital, Affiliated to Shanghai Jiao Tong University, School of Medicine, 639 Zhizaoju Road, Shanghai 200011, China; ^3^Shanghai Jiao Tong University Affiliated Sixth People's Hospital South Branch, 9588 Nanfeng Road, Shanghai 201400, China

## Abstract

Obesity is a clinical syndrome which is driven by interactions between multiple genetic and environmental factors. Monogenic obesity is a rare type of obesity which is caused by a mutation in a single gene. Patients with monogenic obesity may develop early onset of obesity and severe metabolic abnormalities. In this study, we screened mutations of *LEP* in a total of 135 Chinese individuals including 35 obese patients whose BMI ≥32 kg/m^2^ and 100 controls with BMI <25 kg/m^2^. Moreover, detailed information and clinical measurements of the participants were also collected. Finally, we identified a novel nonsynonymous mutation H118L in exon 3 of *LEP* in one patient with BMI 46.0 kg/m^2^. This mutation was not identified in the controls. We speculated that the mutation H118L in *LEP* might be associated with severe obesity in Chinese subjects. However, the substantial mechanism should be further investigated.

## 1. Background

Nowadays, obesity has become an important public health issue, for its prevalence is increasing through the years [1, 2]. Besides, it can also induce severe metabolic abnormalities including type 2 diabetes, hypertension, dyslipidaemia and cardiovascular diseases. It has been confirmed that obesity is influenced by both genetic and environmental factors. Monogenic obesity is a special and rare type of obesity which is caused by a mutation in a single gene and is not affected by the environmental factors. So far, several genes, such as *proopiomelanocortin (POMC)*, *leptin receptor (LEPR)*, *leptin (LEP)*, *proconvertase 1 (PC1)*, and *melanocortin 4 receptor (MC4R)*, have been confirmed as the casual genes to the onset of monogenic obesity [3–10]. *LEP* in humans, for instance, was cloned and identified and in 1995. Human *LEP* locates on chromosome 7q31.3, and its translational product is leptin, which plays a decisive role in the regulation of human appetite and results in severe metabolic disorders [11, 12]. Several mutations in *LEP* have been confirmed to be associated with monogenic obesity [3, 4]. In this study, we aimed to screen the potential mutations in *LEP* in obese patients with BMI ≥32 kg/m^2^ to explore the mechanism of severe obesity in these patients.

## 2. Methods

### 2.1. Ethics Statement

This study was conducted in accordance with the Declaration of Helsinki and approved by the Institutional Review Board of Shanghai Jiao Tong University Affiliated Sixth People's Hospital. Written informed consent was obtained from each participant.

### 2.2. Subjects

35 Chinese Han obese participants with BMI ≥32 kg/m^2^ and 100 Chinese Han control subjects with BMI <25 kg/m^2^ in Shanghai were enrolled in this study. All the participants underwent a detailed clinical investigation. Anthropometric parameters such as body height, body weight, blood pressure, body mass index (BMI), and waist and hip circumference were measured. The clinical characteristics of the two groups are listed in Table 1.

### 2.3. Sequences Analysis

Genomic DNA was extracted from the leukocytes in the peripheral blood samples. The coding region of *LEP* consisted of two fragments and amplified by PCR using the primers designed with the software “Primer Premier 5.” Detailed information of the primers and the products were shown in Table 2. The encoding regions of *LEP* were amplified by a thermal cycler (Veriti, ABI, USA). Then the PCR products were depurated and sequenced directly using the 3130 genetic analyzer (Applied Biosystems, USA). Moreover, we use PolyPhen2 (Polymorphism Phenotyping v2) and SIFT Human Protein to predict the function of the mutation. It divides mutation into several categories such as benign, possibly damaging, or probably damaging on the basis of structure information and functional annotation. PolyPhen2 and SIFT Human Protein are available at http://genetics.bwh.harvard.edu/pph2 and http://sift.jcvi.org/www/SIFT_enst_submit.html, respectively.

## 3. Results

**Figure 1:**
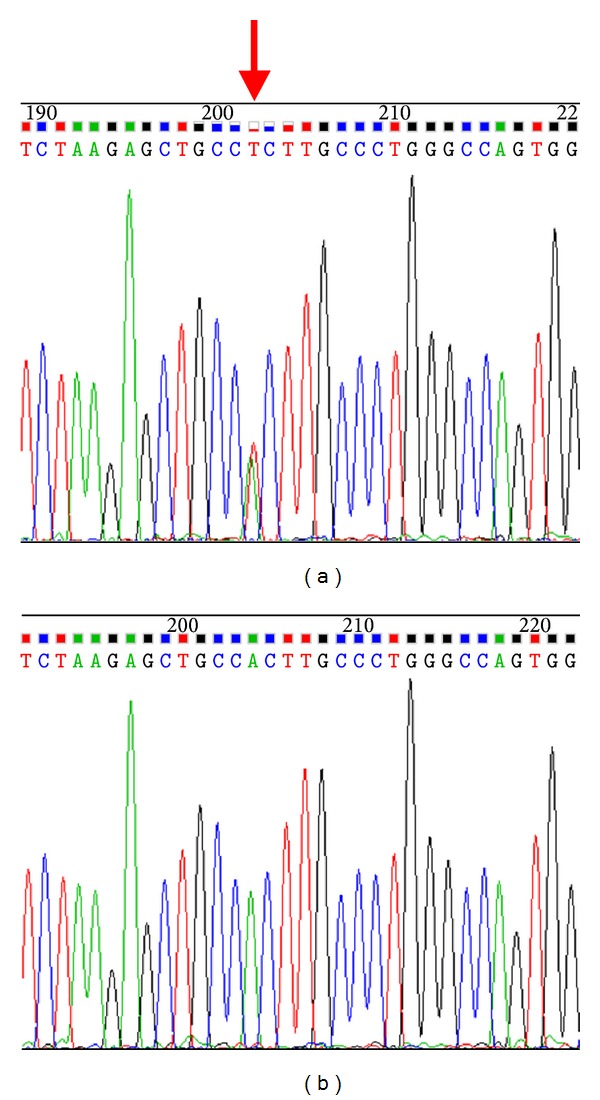
Heterozygous sequence of H118L mutation (a) and normal sequence of *LEP* (b). The red arrow indicated the heterozygous mutation H118L of *LEP*.

After direct sequencing, one novel missense mutation in exon 3 of* LEP* which changed the amino acid from hydrophilic His to hydrophobic Leu (H118L) was detected in one obese patient with BMI 46.0 kg/m^2^ (Figure 1(a)). In addition, his physical examination also showed a typical abdominal obesity appearance, with the waist circumference 145 cm, as well as hip circumference 135 cm. Besides extreme obesity, the carrier also has complications of hypertension, metabolic syndrome, fatty liver syndrome, sleep apnea syndrome, gastric ulcer, and chronic superficial gastritis. However, such mutation was not detected in other obese subjects nor the normal controls (Figure 1(b)). PolyPhen2 showed that the mutation was possibly damaged with a score of 0.041 (sensitivity: 0.94; specificity: 0.83), which is predicted to be benign. In addition, SIFT human protein indicated the mutation to be “DAMAGING.” The two prediction outcomes are also in accordance with the clinical phenotypes of that patient.

## 4. Discussion

*LEP* was expressed in adipose tissue and its product leptin was a classic adipokine, which participated in the food intake and energy expenditure. Therefore, the mutations in *LEP* can possibly damage the function of leptin and disturb the metabolic balance in humans, which is directly responsible for severe obesity and other metabolic disorders. Up to now, several mutations like R105W, N103K, and L72S in *LEP* have been reported to be related with a phenotype of extreme obesity around the world.

In our present study, we screened the whole exons of *LEP* in the obese samples as well as the normal controls in the Chinese and detected a novel mutation H118L in one obese individual but not the normal controls. This novel mutation was not reported in the 1000 Genomes Project neither [13]. Moreover, the missense mutation led to the amino acid residue substitution from His to Leu, which is predicted to be possibly damaged with a score of 0.041 (sensitivity: 0.94; specificity: 0.83) by Polyphen2 and “DAMAGING” by SIFT human protein. Since this mutation carrier exhibited a phenotype of severe obesity with BMI 46.0 kg/m^2^ and complicated with other metabolic disorders including hypertension, metabolic syndrome, fatty liver syndrome, and sleep apnea syndrome, we considered that this mutation might be associated with those abnormal phenotypes.

There are several limitations of our study. Firstly, since the pedigrees and phenotypes of the mutation carrier were not available, whether the mutations were cosegregated with obesity individuals in China or not still remains unknown. In addition, more clinical data such as history of overweight, visceral and subcutaneous fat will be a great help to the certain conclusion. Finally, the functional study of this novel mutation H118L was not carried out to elucidate the mechanism of the disease.

We initially identified a mutation in *LEP* which might be associated with severe obesity in Chinese individuals in our study. However, functional investigations are still needed to confirm our findings and elucidate the mechanism underlying such association between the variants and obesity.

## 5. Conclusion

A novel mutation H118L of *LEP* was detected in the severe obese patient but not the normal controls in the Chinese. We speculated this mutation to be a casual mutation to monogenic obesity in the Chinese. However, further functional studies should be performed to elucidate the substantial mechanism.

## Figures and Tables

**Table 1:** The clinical characteristics of the subjects.

	Cases	Controls
Male/female (*n*)	16/19	47/53
Age (years)	24 (19, 33)	31 (23, 35)
BMI (kg/m^2^)	40.64 ± 7.70	21.58 ± 1.94

Data are expressed as mean ± SD or median (interquartile range).

**Table 2:** Primer Sequences for the amplification.

Name	Forward primer (5′–3′)	Reverse primer (5′–3′)	Product length (bp)	Annealing temperature (°C)
Primer 1	GCCAGAGCAGAAAGCAAA	TCAGGAGGCGTTCAATAA	**397**	**59**
Primer 2	GAGCACTTGTTCTCCCTCTT	TTCCCTTAACGTAGTCCTTG	**435**	**61**
